# Clinicopathological characteristics of immunoglobulin G4-related sialadenitis

**DOI:** 10.1186/s13075-015-0698-y

**Published:** 2015-07-21

**Authors:** Wei Li, Yan Chen, Zhi-Peng Sun, Zhi-Gang Cai, Tong-Tong Li, Lei Zhang, Min-Xian Huang, Hong Hua, Mei Li, Xia Hong, Jia-Zeng Su, Zhu-Yan Zhang, Yan-Ying Liu, Jing He, Zhan-Guo Li, Yan Gao, Guang-Yan Yu

**Affiliations:** Department of Oral and Maxillofacial Surgery, Peking University School and Hospital of Stomatology, Beijing, 100081 China; Department of Oral Pathology, Peking University School and Hospital of Stomatology, Beijing, 100081 China; Department of Oral Radiology, Peking University School and Hospital of Stomatology, Beijing, 100081 China; Department of Oral Medicine, Peking University School and Hospital of Stomatology, Beijing, 100081 China; Department of Nuclear Medicine, Affiliated Beijing Tong Ren Hospital Capital University of Medical Science, Beijing, 100730 China; Department of Rheumatology and Immunology, Peking University People’s Hospital, Beijing, 100044 China

## Abstract

**Introduction:**

Immunoglobulin G4-related disease (IgG4-RD) is a newly recognized fibro-inflammatory condition. Forty-two cases with immunoglobulin G4-related sialadenitis (IgG4-RS) confirmed by histopathological and immunohistochemical assessment were studied to clarify the clinicopathologic characteristics of the salivary glands involved in IgG4-RS, especially the relationship between the histopathologic features and function of salivary glands or serum levels of IgG4.

**Methods:**

Clinical, serologic, imaging and histopathological data of these cases were analyzed. CT volumes of submandibular, parotid, and lacrimal glands were calculated. The saliva flow rate was measured. Scintigraphy with 99mTc-pertechnetate was undertaken in 31 cases, and the concentration index (CI) and secretion index (SI) was calculated. Relationships between fibrosis severity and salivary gland function or serum IgG4 levels were analyzed.

**Results:**

The first symptom was swelling of bilateral submandibular or lacrimal glands. Physical examination showed multiple bilateral major salivary glands (including sublingual and accessory parotid glands) and lacrimal glands were enlarged in IgG4 RS. Multiple enlarged cervical lymph nodes were noted in 30 patients. Saliva flow at rest was lower than normal in 34 cases; stimulated saliva flow was lower than normal in 15 cases. Secretory function was reduced more severely in the submandibular glands than in the parotid glands. Serum levels of IgG4 were elevated in 95.2% of cases and 78.6% patients had increased IgE levels. Serum IgG4 level was higher and saliva secretion lower as glandular fibrosis increased.

**Conclusions:**

Prominent changes in the morphology, histology, immunohistochemistry and secretion of the major salivary glands of IgG4-RS patients were accompanied by involvement of the lacrimal glands and cervical lymph nodes. Elevated IgE, allergic history, eosinophil infiltration suggest allergic reactions as a potential pathogenesis of IgG4-RS. Severity of glandular fibrosis correlated with salivary function and serum levels of IgG4.

## Introduction

Immunoglobulin G4-related disease (IgG4-RD) is a newly recognized fibroinflammatory condition. The disease is characterized by tumefactive lesions, a dense lymphoplasmacytic infiltrate rich in IgG4-positive plasma cells, storiform fibrosis, and often (but not always) elevated serum concentrations of IgG4 in organs [1]. IgG4-RD was not recognized as a systemic disease until 2003, when extrapancreatic manifestations were identified in patients with autoimmune pancreatitis (AIP) [2].

IgG4-RD has been described in virtually every organ system: the biliary tree, salivary glands, periorbital tissues, kidneys, lungs, lymph nodes, meninges, aorta, breast, prostate gland, thyroid gland, pericardium, and skin [2–6]. Symptoms vary depending on the affected organs. Some patients may experience severe complications, such as obstruction or compression symptoms due to organomegaly or hypertrophy, as well as organ dysfunction caused by cellular infiltration or fibrosis [7].

Identification of IgG4-positive plasma cells in Küttner tumors and Mikulicz disease propelled renewed interest in these diseases, and fueled re-analyses of the classification of inflamed salivary glands [8–10]. Considerable evidence supports the concept of IgG4-related sialadenitis (IgG4-RS), a category that subsumes Küttner tumors and Mikulicz disease.

Since IgG4-RS has been established as an entity in this century, clinicopathologic studies have been carried out in different countries [8, 9, 11, 12]. The disease seems to be found worldwide. However, the clinicopathologic features of IgG4-RS are not well characterized. A few studies on functional changes compared with the histopathologic features of the involved salivary gland have been reported [13]. Finding sufficient histopathologic information on IgG4-RS for pathologic grading is difficult owing to a lack of biopsy materials.

The present study was carried out to obtain more clinicopathologic information about IgG4-RS. In particular, we wished to clarify the characteristics of the salivary gland(s) involved in IgG4-RS. The relationship between the histopathologic features and function of salivary glands, the serum level of IgG4, and the number of IgG4-positive cells were analyzed to judge disease severity.

## Methods

The study protocol was approved by the Ethical Committee for Human Experiments of the Peking University School of Stomatology (Beijing, China) and was conducted in accordance with the Declaration of Helsinki guidelines for human research. All patients provided informed consent prior to participation in this study.

Forty-two patients were referred to the Department of Oral and Maxillofacial Surgery at Peking University School of Stomatology between August 2011 and June 2014. The patients were selected for this study because of bilateral swelling of submandibular glands with or without swelling of multiple exocrine glands (lacrimal, parotid, and sublingual glands) of >3 months duration. The diagnosis was made on the basis of symptoms, serologic analyses, imaging, and histopathological findings [14]. Clinical data (age, sex, anatomic site, duration of swelling, imaging, and physical examination) were recorded.

### Measurement of the volume of major salivary glands and lacrimal glands

Computed tomography (CT) was carried out with an eight-slice scanner (BrightSpeed; GE Medical Systems, Piscataway, NJ, USA). Volumes of the lacrimal, parotid, and submandibular glands were reconstructed by volume rendering [15, 16] and compared with normal values that we provided previously [17]. The diagnosis of glandular enlargement was made if their volumes were larger than normal values. The accessory parotid glands are only pea-sized upon CT imaging. Enlargement of the accessory parotid glands was judged if CT showed a volume obviously larger than 1 cm in diameter. Judgment of enlargement of the sublingual glands was by physical examination because of the difficulty in measurement by imaging. CT images were assessed by an experienced radiologist and a maxillofacial surgeon (Z-PS and WL).

### Measurement of salivary secretion

Whole saliva was collected as described previously [18, 19]. Five minutes after collection of whole saliva at rest, stimulated whole saliva using 2.5 % citric acid solution was collected for another 5 minutes. The saliva flow rate was calculated using the following formula:

$$ \mathrm{Saliva}\kern0.5em \mathrm{flow}\kern0.5em \mathrm{rate}=\left(\mathrm{weight}\;\mathrm{of}\;\mathrm{tube}\;\mathrm{with}\;\mathrm{collected}\;\mathrm{saliva}\;\hbox{--}\;\mathrm{weight}\;\mathrm{of}\;\mathrm{empty}\;\mathrm{tube}\right). $$

### Scintigraphy

Scintigraphy with ^99m^Tc-pertechnetate was undertaken in 31 cases using a standardized protocol [20]. The concentration index (CI) was calculated using the following formula:

$$ \mathrm{C}\mathrm{I}=\left(\mathrm{maximum}\;\mathrm{uptake}\;\mathrm{value}\;\hbox{--}\;\mathrm{background}\right)/\ \mathrm{background}. $$

The period from stimulation using 2.5 % citric acid solution to the minimum value after stimulation within 30 minutes was considered to be the “secretion phase”. The secretion index (SI) was calculated using the following formula:

$$ \begin{array}{c}\hfill \mathrm{S}\mathrm{I}=\Big(\mathrm{maximum}\;\mathrm{value}\;\mathrm{before}\;\mathrm{stimulation}\;\mathrm{using}\;\mathrm{citric}\;\mathrm{acid}\;\hbox{--}\;\mathrm{minimum}\;\mathrm{value}\;\mathrm{after}\;\mathrm{stimulation}\;\mathrm{using}\;\mathrm{citric}\hfill \\ {}\hfill \mathrm{acid}\Big)/\left(\mathrm{maximum}\;\mathrm{value}\;\mathrm{before}\;\mathrm{stimulation}\;\mathrm{using}\;\mathrm{citric}\;\mathrm{acid}\;\hbox{--}\;\mathrm{background}\right)\times 100\ \%.\hfill \end{array} $$

Mean values of bilateral sides were taken to be the CI and SI of the parotid and submandibular glands, respectively.

### Serologic examinations

Serologic analyses were undertaken for antinuclear antibody (ANA), IgE, IgA, IgM, total IgG, and subclasses of IgG using the Array 360 Immunoassay Assay Protein Serology Chemistry Analyzer system (Beckman Coulter, Fullerton, CA, USA) by rate nephelometry immunoassay after generating charts and logs from quality control materials and the quality control. Anti-SS-A and anti-SS-B antibodies were tested by western blotting.

### Histological examination

All 42 cases underwent histopathologic examination. Hematoxylin and eosin-stained slides of all cases were reviewed by two pathologists (YC and YG) independently according to the diagnostic criteria for IgG4-RS [7, 14]. Three high-power fields (HPFs) with the greatest density of eosinophils were quantified (HPF area = 0.2375 mm^2^).

The severity of inflammation and fibrosis was divided into three stages according to Seifert’s classification with slight modification [21]. The first stage proposed by Seifert was abandoned because focal chronic inflammation with nests of lymphocytic infiltrates was not sufficient to diagnose IgG4-RS by pathologic means.

**Fig. 1:**
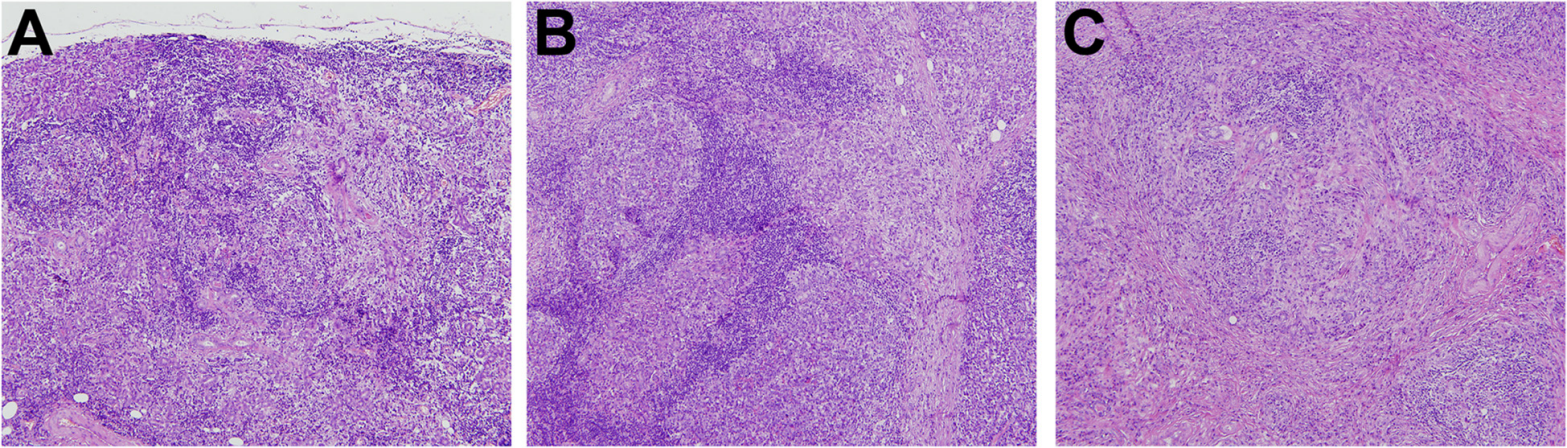
The inflammation and fibrosis grade of IgG4-RS. **a** Stage 1: marked diffuse lymphoplasmacytic infiltration. Lymphoid follicles have developed. Fibrosis in the centers of lobules or periductal areas and atrophy of acini (H&E staining, ×100 magnification). **b** Stage 2: even more prominent lymphoplasmacytic infiltration, with formation of lymphoid follicles with reactive germinal centers, reduction of the parenchyma in the salivary gland, myofibroblast hyperplasia, and storiform fibrosis. Severe fibrosis in lobule centers or periductal areas as well as severe atrophy of acini, but the outline of lobules is retained (H&E, ×100). **c** Stage 3: destruction of the lobular architecture with marked parenchymal loss and sclerosis, the outlines of lobules are not clear. Germinal centers have degenerated and the light area is obscured (H&E, ×100). *H&E* hematoxylin and eosin

Stage 1 referred to marked diffuse lymphoplasmacytic infiltration. Lymphoid follicles had developed. Fibrosis in the centers of lobules or periductal areas as well as atrophy of acini were noted (Fig. 1a). Stage 2 referred to even more prominent lymphoplasmacytic infiltration: formation of lymphoid follicles with expanded reactive germinal centers, reduction of the parenchyma of secretory glands, myofibroblast hyperplasia, and storiform fibrosis. One could observe severe fibrosis in the centers of lobules or periductal areas as well as severe atrophy of acini, but the outlines of the lobules remained (Fig. 1b). Stage 3 referred to destruction of the lobular architecture with marked parenchymal loss and sclerosis: the outlines of lobules were not clear. Germinal centers degenerated and light areas were obscured (Fig. 1c).

### Immunohistochemical examination

Tissue sections (thickness 4 μm) were deparaffinized and rehydrated. Endogenous peroxidase activity was blocked using 3 % H_2_O_2_. For antibodies against human IgG4 (Epitomics, Burlingame, CA, USA), IgG (Epitomics), and CD21 (Zhongshan, Beijing, China), antigen retrieval was achieved by treating sections with a boiling citric acid buffer solution (0.01 mol/l, pH 6.0) for 10 minutes in a microwave oven. Color was developed in freshly made diaminobenzidine. Sections were washed briefly in running tap water and stained lightly with Mayer’s hematoxylin. Negative controls were obtained by omission of the relevant primary antibody.

Three identical HPFs with the greatest density of IgG-positive and IgG4-positive plasma cells were quantified. The ratio of IgG4-positive plasma cells to IgG-positive plasma cells was calculated for each patient.

### Statistical analyses

Statistical analyses were performed using SPSS version 13.0 (SPSS, Chicago, IL, USA). Categorical variables were compared using the chi-square or Fisher exact test. Continuous variables are the mean ± standard deviation, and compared by the independent *t* test or the Wilcoxon rank sum test. *P* <0.05 was considered significant.

## Results

Of the 42 patients who comprised the study cohort, 16 were males and 26 were females with a ratio of 1:1.625. The median age was 55 (range 23–89) years.

### Case history

The first sign that patients found was bilateral swelling of the submandibular glands (12/42, 28.6 %) or the lacrimal glands (14/42, 33.3 %), followed by swelling of the unilateral submandibular gland (5/42, 11.9 %), bilateral parotid gland (5/42, 11.9 %), and bilateral sublingual gland (1/42, 2.4 %). The interval between swelling of the first gland and swelling of the second gland was 1–96 (mean 22.6) months. Four (9.5 %) patients had AIP, six (14.3 %) cases had asthma, six (14.3 %) subjects had interstitial pneumonia, 22 (52.4 %) cases had rhinosinusitis, and two (4.8 %) cases had dysaudia.

### Physical examination

**Fig. 2:**
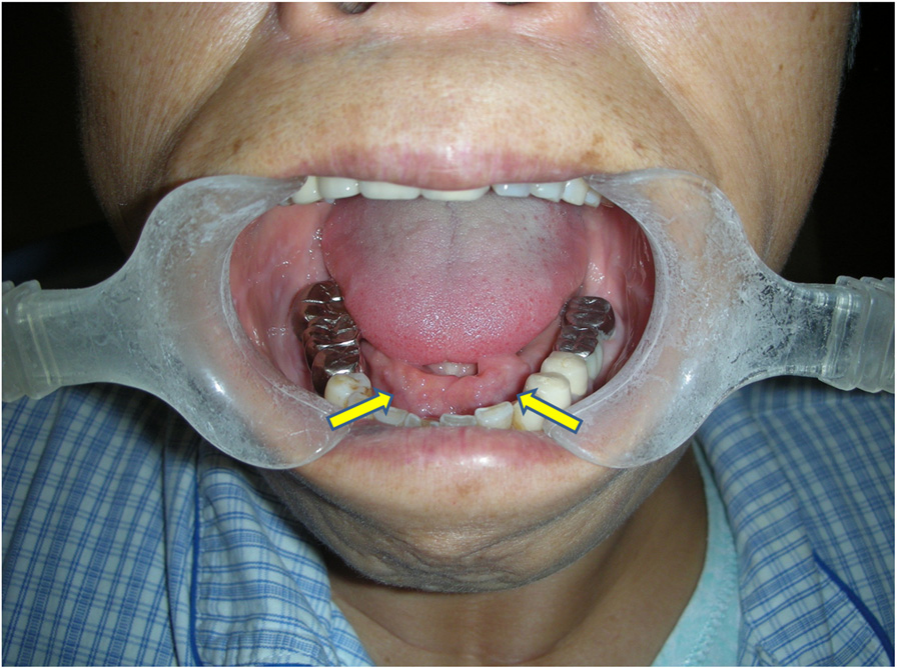
Enlargement of the sublingual glands (*arrows*)

**Fig. 3:**
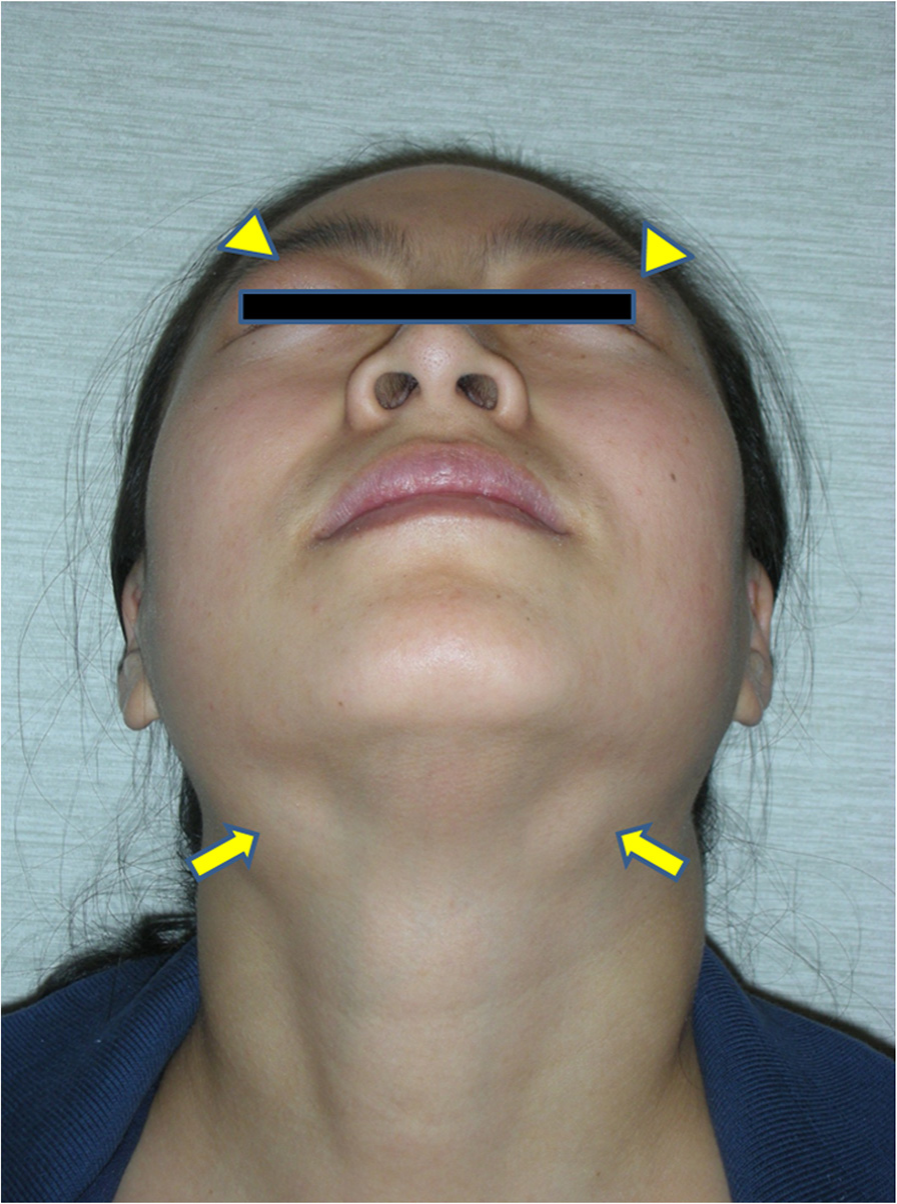
Enlargement of the lacrimal (*arrowheads*) and submandibular glands (*arrows*)

Physical examinations revealed that 64 of the total 69 submandibular glands (15 submandibular glands were resected before physical examination) were enlarged in 27 bilateral cases and 10 unilateral cases. Twenty-two out of 83 parotid glands (one parotid gland was resected before physical examination) were enlarged in nine bilateral cases and four unilateral cases. Fifty-five lacrimal glands were enlarged in 27 bilateral cases and one unilateral case. Bilateral enlargement of sublingual glands was observed in 22 patients (Fig. 2). Bilateral enlargement of the accessory parotid glands was noted in eight patients. Enlargement of at least two major salivary glands or lacrimal glands was found in 35/42 (83.3 %) patients (Fig. 3). Multiple enlarged cervical lymph nodes were observed in 30 patients.

Upon milking the submandibular or parotid glands, a reduced amount of clear saliva flowed out of the ductal orifice in 26 patients. No obvious saliva could be milked from the submandibular gland in 15 (35.7 %) cases or from the parotid gland in five patients. The oral mucosa was wet with preservation of the saliva pool at the mouth floor in 28 patients. Mild dryness of the oral mucosa was found in seven (16.7 %) cases but the saliva pool had disappeared in 14 (33.3 %) cases. Rampant caries was not observed.

### CT volumes of submandibular, parotid, and lacrimal glands

**Fig. 4:**
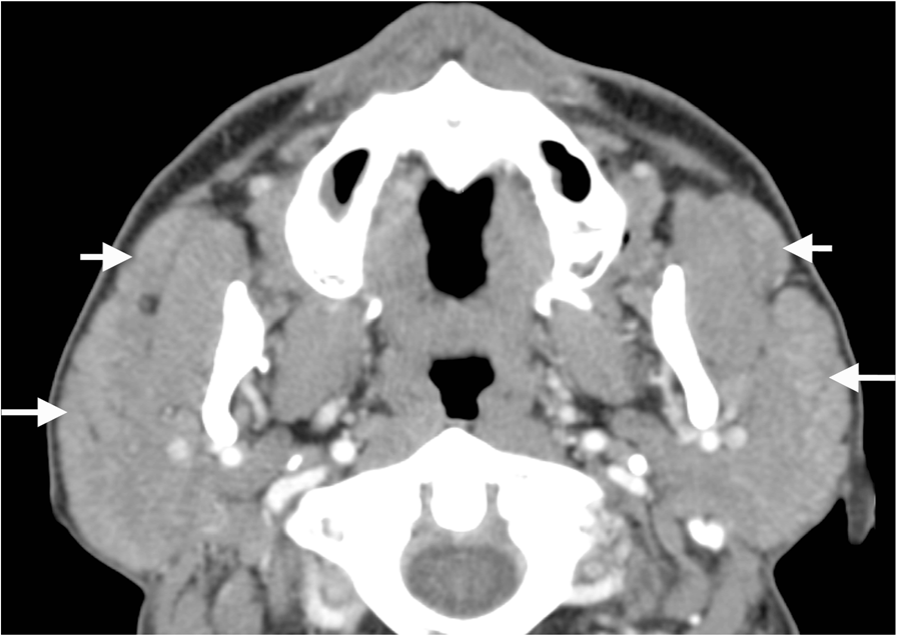
Bilateral enlargement of the accessory parotid glands (*short arrows*) and main parotid glands (*long arrows*) are observed on CT film

Forty-one patients underwent CT examination. CT volumes of the enlarged submandibular, parotid and lacrimal glands are presented in Table 1. Compared with the normal reference of glandular volumes, 67 out of 69 submandibular glands (13 submandibular glands were resected before CT examination) were enlarged in 30 bilateral cases and seven unilateral cases. Fifty-three out of 81 parotid glands (one parotid gland was resected before CT examination) were enlarged in 24 bilateral cases and five unilateral cases. Fifty-nine out of 79 lacrimal glands (one lacrimal gland was resected before CT examination and one patient did not undergo CT examination of the lacrimal glands) were shown to be enlarged in 27 bilateral cases and five unilateral cases. Bilateral enlargement of the accessory parotid glands was found in 9/41 (22.0 %) cases (Fig. 4).

**Table 1:** Quantitative measurements of the volumes of enlarged glands in IgG4-related sialadenitis and normal controls

Age (years)	Parotid gland						Submandibular gland						Lacrimal gland					
	Male			Female			Male			Female			Male			Female		
	Normal (*n* = 90)	IgG4-RS (*n* = 19)	*P*	Normal (*n* = 90)	IgG4-RS (*n* = 36)	*P*	Normal (*n* = 90)	IgG4-RS (*n* = 22)	*P*	Normal (*n* = 90)	IgG4-RS (*n* = 45)	*P*	Normal (*n* = 90)	IgG4-RS (*n* = 21)	*P*	Normal (*n* = 90)	IgG4-RS (*n* = 38)	*P*
22–44	22.187 ± 6.070	29.400 ± 0.879	†	19.612 ± 4.257	28.237 ± 3.119	†	NS	NS	NS	7.310 ± 2.282	15.034 ± 5.109	†	0.432 ± 0.164	3.745 ± 21.280	†	0.401 ± 0.134	2.264 ± 0.526	†
45–59	31.880 ± 9.061	42.242 ± 7.227	†	21.031 ± 4.033	38.061 ± 10.214	†	8.637 ± 2.454	17.406 ± 4.222	†	7.945 ± 1.847	14.492 ± 3.866	†	0.667 ± 0.217	2.103 ± 0.714	†	0.624 ± 0.154	2.256 ± 1.236	†
≥60	NS	NS	NS	31.846 ± 8.560	45.235 ± 8.468	†	9.410 ± 3.076	14.507 ± 1.562	†	7.603 ± 1.678	12.037 ± 3.054	†	0.549 ± 0.166	0.874 ± 0.176	†	0.483 ± 0.113	1.443 ± 0.828	†

Data presented as mean ± standard deviation (cm^3^)

*IgG4-RS* IgG4-related sialadenitis, *NS* not specified

† *P* <0.01

### Flow rate of whole saliva and scintigraphy

The saliva flow rate at rest was 0.07–4.88 (mean 1.02 ± 0.94) g/5 minutes. Flow rates were lower than the normal value (1.9 g/5 minutes) in 34 (81.0 %) cases. The stimulated saliva flow rate was 2.06–17.34 (mean 8.76 ± 4.10) g/5 minutes, and nine (35.7 %) cases showed a lower stimulated saliva flow rate than the normal value (6.75 g/5 minutes) [22].

Thirty-one cases underwent ^99m^Tc-pertechnetate scintigraphy to evaluate the uptake and secretory function of submandibular and parotid glands. The mean CI of the submandibular glands was lower than normal value in 22.6 % patients, whereas the mean CI of the parotid glands was normal for all patients. The mean SI of the submandibular glands was lower than the normal value in 77.4 % patients, whereas the mean SI of the parotid glands was lower than the normal value in 45.2 % of subjects. Secretory function was reduced more severely in the submandibular glands than in the parotid glands.

### Serologic examinations

In the 39 cases in whom the percentage of eosinophils in peripheral blood was measured, eight (20.5 %) cases showed elevated levels (7.7–14.1 %). The level of IgG was elevated in 53.8 % of patients. The level of IgG4 was elevated in 95.2 % of subjects. A total of 28.6 % of subjects had an elevated level of IgG2, and 78.6 % of patients had an increased level of IgE. The reaction to anti-SSA, anti-SSB, and anti-ANA was negative in all patients except for one patient who had a positive reaction to anti-SSA.

### Histopathological and immunohistochemical examinations

Gross views revealed the involved glands to be enlarged and firm but their configurations to be maintained. The cut surface varied in different areas. In the most serious areas, gland tissue was homogeneous and appeared as an ill-defined mass. The color was white or pale yellow. Upon more careful study, the structure of glandular lobules could be ascertained. Uninvolved areas retained the appearance of a normal gland.

Histological sections revealed preservation of the lobular architecture, marked lymphoplasmacytic inflammation, large irregular lymphoid follicles with expanded germinal centers, and acinar atrophy without prominent lymphoepithelial lesions. Characteristically, prominent cellular interlobular fibrosis was shown to be due to activated fibroblasts, and infiltration of lymphocytes and plasma cells was shown. Plasma cells were mature without sign of atypia. Eosinophil infiltration in the involved areas was noted in 20 cases (47.6 %), and the number ranged from 2/HPF to 98/HPF (median 16/HPF). Obliterative phlebitis was noted in 30 cases.

According to our modified Seifert classification, out of 42 cases 14 patients were classified as stage-1 disease, stage-2 disease, and stage-3 disease, respectively.

Numbers of IgG4-positive-stained plasma cells were increased in all cases. Mean numbers of IgG-positive and IgG4-positive plasma cells per HPF were 167 ± 42 (range 79–246) and 124 ± 42 (range 56–215), respectively. The IgG4/IgG ratio was 73.7 ± 12.8 % (range 50.4–93.9 %).

### Relationship between histopathological changes and clinical features

Clinical features, serologic and immunohistochemical examinations, and secretion function of the salivary glands of patients with stage-1, stage-2 and stage-3 diseases are summarized in Table 2. Serum levels of IgG and IgG4 as well as the numbers of IgG-positive and IgG4-positive cells increased, whereas the ratio of (IgG1 + IgG3) / (IgG2 + IgG4) decreased significantly (*P* <0.05) as glandular fibrosis became more severe. Also, the stimulated flow rate and CI of the submandibular gland decreased significantly (*P* <0.05) as glandular fibrosis became more severe.

**Table 2:** Relationship between different parameters and histopathologic grade

			Histopathologic grade			
			I	II	III	*P*
			(*n* = 14)	(*n* = 14)	(*n* = 14)	
Clinical features						
	Male/female		3: 4	3: 11	1:1	
	Age (years)		54.1	52.0	57.4	0.539
	Volumes of swollen salivary glands	PG	39.65 ± 8.15	38.68 ± 10.9	34.56 ± 6.03	0.223
		SMG	14.52 ± 2.00	16.31 ± 3.40	16.23 ± 2.64	0.068
Serologic examinations						
	IgG (mg/dl)		15776.4 ± 6304.9	15838.0 ± 3993.0	26226.7 ± 9820.8	0.001
	IgG2 (mg/dl)		539.6 ± 225.3	510.8 ± 195.2	531.7 ± 221.8	0.935
	IgG4 (mg/dl)		647.8 ± 700.1	818.6 ± 894.1	1802.8 ± 1011.0	0.002
	T-IgE (KU/U)		626.4 ± 705.5	361.9 ± 401.8	372.2 ± 344.2	0.433
	EOS (%)		4.7 ± 3.3	3.6 ± 3.4	4.8 ± 3.6	0.638
	(IgG1 + IgG3) / (IgG2 + IgG4) (%)		88.3 ± 48.7	70.6 ± 23.0	47.2 ± 17.5	0.007
Immunohistochemical examinations	IgG4^+^ (cells/HPF)		90 ± 27	134 ± 33	143 ± 43	0.001
	IgG^+^ (cells/HPF)		133 ± 37	172 ± 37	191 ± 42	0.001
	IgG4^+^/IgG^+^ (%)		68.7 ± 12.2	78.0 ± 9.8	74.0 ± 13.2	0.125
	EOS (cells/HPF)		23 ± 22	17 ± 7	11 ± 7	0.097
Flow rate of whole saliva	Rest (g/5 minutes)		1.04 ± 0.72	1.04 ± 1.27	0.82 ± 0.51	0.757
	Stimulated (g/5 minutes)		10.11 ± 3.23	8.12 ± 4.24	6.78 ± 2.57	0.045
Scintigraphy	CI (%)	PG	74.22 ± 9.12	77.02 ± 10.77	67.26 ± 11.00	0.094
		SMG	53.29 ± 12.54	49.05 ± 9.10	34.20 ± 9.26	<0.001
	SI (%)	PG	47.33 ± 15.10	52.14 ± 10.15	46.90 ± 14.50	0.603
		SMG	38.50 ± 17.50	27.12 ± 9.70	26.1 ± 8.50	0.055

*CI* concentration index, *EOS* eosinophil, *HPF* high power field, *PG* parotid gland, *SI* secretion index, *SMG* submandibular gland, *T-IgE* total immunoglobulin E

## Discussion

IgG4-RD is accepted as an immune-mediated disease involving multiple disorders such as sclerosing pancreatitis, sclerosing cholangitis, tubulointerstitial nephritis, interstitial pneumonia, retroperitoneal fibrosis, inflammatory pseudotumor of the liver and lung, inflammatory aortic aneurysms, and enlargement of the salivary and lacrimal glands [2, 23–25]. IgG4-RS is a component of IgG4-RD, and the main presentation is enlargement of the salivary and lacrimal glands.

Histopathological studies based on biopsy material involving IgG4-RS in the major salivary glands are few. Most histopathological information is derived from whole-gland ectomy specimens. We believe it is important to take biopsies from the involved major salivary gland (especially the submandibular glands) to make a definite diagnosis of IgG4-RS for two main reasons. First, a relatively large dose of glucocorticoids (“assault therapy”) in the short term and a small dose as maintenance therapy in the long term are typical first-line therapies [1]. The results are excellent, but the side effects can limit use of these regimens. Such treatments should therefore be given only to patients with a definitive diagnosis of IgG4-RS. Second, an elevated serum level of IgG4 is found not only in IgG4-RD, but also in Churg–Strauss syndrome, multicentric Castleman disease, and eosinophilic disorders as well as in some patients with rheumatoid arthritis, systemic sclerosis, chronic hepatitis, and liver cirrhosis [26, 27]. Sah and Chari [27] found that approximately 30 % of patients had a normal serum IgG4 level despite typical histopathological and immunohistochemical findings of IgG4-RD. In the present series, two cases had a normal serum level of IgG4 despite also possessing the typical clinicopathologic features of IgG4-RS. In contrast, the histopathological and immunohistochemical appearances did not meet the diagnostic criteria of IgG4-RS even though the serum level of IgG4 was elevated in two cases (who were excluded from our study). Histopathological and immunohistochemical analyses of biopsy specimens therefore remain the cornerstone for the diagnosis of IgG4-RS.

The relatively superficial location of the submandibular glands enables incisional biopsy, which is difficult for affected internal organs such as the pancreas and kidneys. The biopsy was undertaken under local anesthesia. The superficial part of the glands was chosen for biopsy. Gland tissue of dimension 0.7 cm × 0.5 cm was excised for biopsy. The wound in the glands was closed carefully. No complications were found and secretory function was not affected.

Usually, IgG4-RS involves swelling of multiple exocrine glands (including lacrimal, parotid, and submandibular glands). This was true of our series. Gland volume was measured by CT volume rendering and was compared with normal values. Compared with the results of palpation, the number of enlarged parotid glands increased obviously according to the measurement of gland volume by CT. Twenty-two out of 83 parotid glands were enlarged in nine bilateral cases and four unilateral cases according to physical examination. Fifty-three of 81 (one patient did not undergo CT examination) parotid glands were enlarged in 24 bilateral cases and five unilateral cases according to measurement of the gland volume by CT. The main reason for these discrepancies between the two methods of measurement is that the anatomy of the parotid glands is irregular and difficult for accurate evaluation by palpation if the swelling is not obvious, whereas CT volume rendering can detect enlargement of the parotid glands accurately and quantitatively. The high prevalence of enlargement of the parotid glands in IgG4-RS should be noted, and CT volume rendering is advocated. Bilateral enlargement of the sublingual glands and accessory parotid glands is very rare in other diseases but is relatively common in IgG4-RS. In the present study, unexpected bilateral swelling of the sublingual glands (52.4 %) and accessory parotid glands (22.0 %) was observed. Bilateral swelling of the sublingual glands and/or accessory parotid glands is therefore an important clinical feature in the diagnosis of IgG4-RS.

We agree with Geyer and Deshpande [28] that unilateral and solitary gland involvement should also be accepted within the spectrum of IgG4-RS. In the present study, the mean interval between the first swelling of glands and subsequent swelling of glands was 22.6 months. Cases involving a unilateral gland could be followed by involvement of more glands if treatment is not given.

As a component of IgG4-related systemic disease, careful monitoring of the pancreas, gallbladder, kidneys, and lungs should be undertaken to identify other systemic diseases in the diagnosis of IgG4-RS. The nose, paranasal sinuses, and ears are also involved very often along with the salivary and lacrimal glands in IgG4-RS. A total of 71.4 % cases in the present study had lymphadenopathy of the upper cervical lymph nodes. Swelling of the cervical lymph nodes was the first symptom and sign in three cases. Lymphadenopathy of the cervical lymph nodes should therefore also be considered an important clinical feature in the diagnosis of IgG4-RS.

Saliva flow rates and scintigraphy with ^99m^Tc-pertechnetate are effective for evaluation of the function of the major salivary glands. These procedures are relatively easy to carry out, noninvasive, and well tolerated. Studies have shown that IgG4-RS leads to a mild reduction in saliva flow [29]. In the present study, most cases showed a mild or moderate reduction in the saliva flow rate. The saliva flow rate at rest was lower than the normal value in 81.0 % of cases and the stimulated flow rate was lower than the normal value in 35.7 % of subjects. ^99m^Tc-pertechnetate scintigraphy showed that the percentage of patients with a mean SI lower than the normal value was higher in the submandibular glands (77.4 %) than in the parotid glands (45.2 %), suggesting that damage to the submandibular glands was more severe than that to the parotid glands in IgG4-RS.

There is evidence of autoimmune disorder in AIP but there are also signs of an allergic nature in its pathogenesis. In our series, an elevated serum level of IgG4 was detected in 95.2 % of cases along with an obviously elevated level of IgE in 78.6 %. In one study of 42 patients with AIP, the IgG4 level was elevated in 93 % of cases and the IgE level was elevated in 86 % of cases [30]. High levels of IgE have also been detected in IgG4-related lymphadenopathy and IgG4-related kidney disease [31]. In the present study, 20.5 % of patients had an increased percentage of eosinophils in peripheral blood. Histological evaluation revealed eosinophil infiltration in inflamed glands in 47.6 % of cases. Asthma was observed in 14.3 % of IgG4-RS cases. These results suggest a possible allergic origin of IgG4-RS.

Elevated IgE levels and eosinophil infiltration are among the criteria for the diagnosis of IgG4-RD [14]. Serum concentrations of IgG4 and IgE have been shown to be significantly higher in IgG4-RD than in Sjögren syndrome [24]. However, few studies on IgG4-RS have focused on serum concentrations of IgE and eosinophil infiltration [32, 33]. We propose that elevated serum levels of IgE, history of allergic diseases, and eosinophil infiltration should be taken into consideration in the diagnosis of IgG4-RS.

Seifert [21] put forward a histopathologic grading standard for chronic sclerosing sialadenitis. That classification was based on inflammation of the salivary glands (including some local factors that induced salivary inflammation) without the prominent characteristics of autoimmune systemic disease. Ectopic germinal centers and fibroblasts are the main causes of the development and progression of autoimmune inflammation [34–36]. To accord with the histopathologic characteristics of IgG4-RS, the Seifert classification was modified into three grades on the basis of germinal centers and fibrosis in our study. This grading system can reflect the severity of fibrosis in the salivary glands.

As the severity of glandular fibrosis increased, serum levels of IgG and IgG4 in the three classes increased significantly, whereas the (IgG1 + IgG3) / (IgG2 + IgG4) ratio decreased significantly. These data suggest that the results of serological examinations have a good correlation with histopathologic grading, thereby enabling serological results to be used to estimate lesion severity.

The stimulated flow rate of whole saliva decreased with increasing histopathological grade. Increased fibrosis was accompanied by a decreased CI in scintigrams in the submandibular glands, suggesting that the increase in inflammatory fibrosis and glandular function had declined. Treatment of IgG4-RS should therefore be initiated as early as possible once the definitive diagnosis is established to preserve the function of involved salivary glands.

## Conclusions

Biopsy is very important to provide the necessary information for the definitive diagnosis of IgG4-RS. Bilateral swelling of the sublingual glands and accessory parotid glands, lymphadenopathy of the cervical lymph nodes, and bilateral enlargement of parotid and submandibular glands are important clinical features of IgG4-RS. Elevated serum levels of IgE, allergic history, and eosinophil infiltration suggest allergic reactions as a potential pathogenesis. Increased fibrosis accompanied with lower salivary function suggests that the early diagnosis and treatment of IgG4-RS is very important.

## Abbreviations

AIP: Autoimmune pancreatitis
ANA: Antinuclear antibody
CI: Concentration index
CT: Computed tomography
HPF: High-power field
IgG4-RD: Immunoglobulin G4-related disease
IgG4-RS: Immunoglobulin G4-related sialadenitis
SI: Secretion index
